# CAR-T Therapy in Lymphoma Patients With Coexisting Cardiomyopathy or Cardiac Lymphomatous Involvement

**DOI:** 10.1016/j.jaccas.2023.101840

**Published:** 2023-04-21

**Authors:** Choon Ta Ng, Hilda M. Gonzalez Bonilla, Ian Chang, M. Tun Aung, Jennifer J. Gile, Naveen L. Pereira, Jose C. Villasboas Bisneto, Patrick B. Johnston, Hector R. Villarraga, Martin G. Rodriguez-Porcel, Grace Lin, Yi Lin, Joerg Herrmann

**Affiliations:** a Department of Cardiovascular Medicine, Mayo Clinic Rochester, Rochester, Minnesota, USA; b Department of Cardiology, National Heart Centre Singapore; c Department of Hematology, The University of Kansas Cancer Center, Kansas City, Kansas, USA; d Department of Hematology, Mayo Clinic Rochester, Rochester, Minnesota, USA

**Keywords:** CAR-T, cardiac, cardio-oncology, cardiomyopathy, lymphoma, metastasis

## Abstract

Chimeric antigen receptor T-cell (CAR-T) therapy has revolutionized the management of aggressive hematologic malignancies. However, its role in patients with lymphoma and cardiac metastasis or cardiomyopathy remains undefined due to potentially life-threatening complications such as ventricular rupture, cardiac tamponade, and circulatory failure. We present a case series of patients with lymphoma and cardiomyopathy or cardiac metastasis managed with chimeric antigen receptor T-cell therapy. (**Level of Difficulty: Advanced.**)

## Central Illustration

**Figure:**
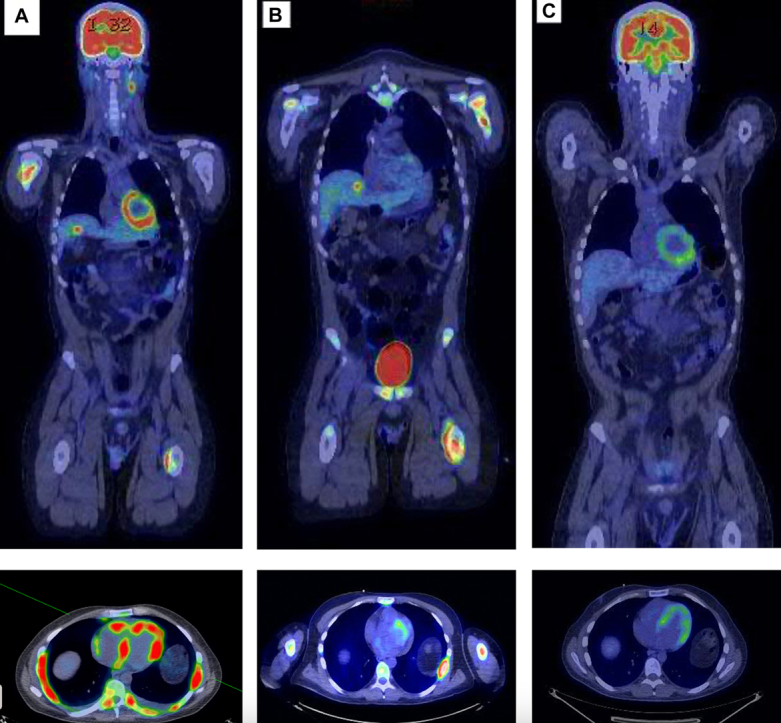

Chimeric antigen receptor T-cell (CAR-T) therapy, which involves the infusion of genetically engineered autologous T cells, has improved the management of patients with refractory or relapsed hematologic malignancies. However, evidence for CAR-T therapy in patients with lymphoma and cardiomyopathy or cardiac metastasis is lacking as these subjects were excluded from the seminal clinical trials. 1, 2, 3 Herein, we summarize our experience with the use of CAR-T therapy in these challenging cases. Key aspects of the cases are presented in the following sections; further details on the chemotherapy regimens and complications of CAR-T therapy are summarized in Supplemental Table 1.

**Learning Objectives**

• To recall and recognize potential cardiac complications associated with CAR-T therapy such as CRS.
• To understand the impact of pre-existing cardiomyopathy or cardiac involvement in patients with lymphoma undergoing CAR-T therapy.
• To define the role of CAR-T therapy in patients with lymphoma and cardiac metastasis or pre-existing cardiomyopathy.

## Patient 1

**Figure 1:**
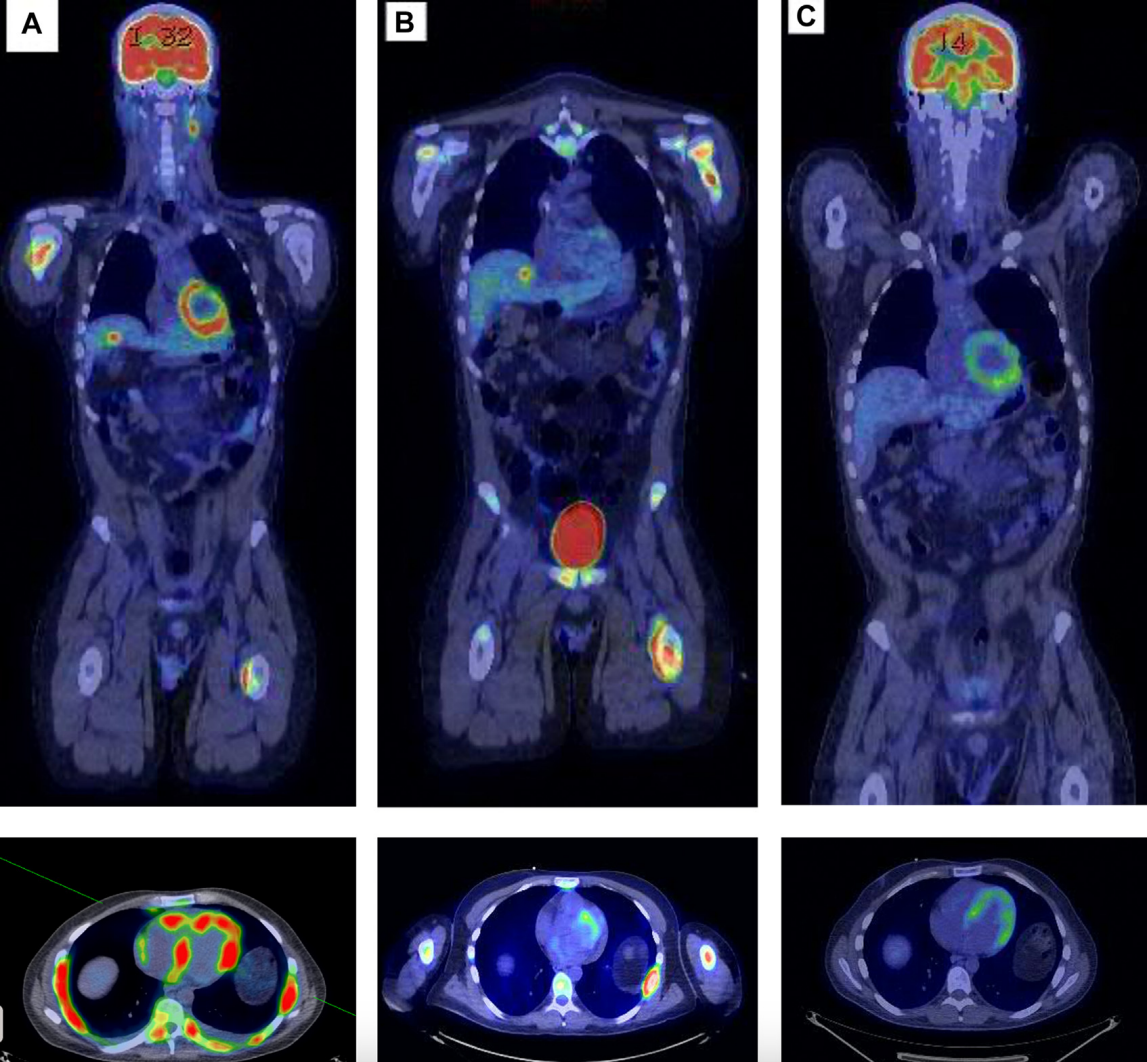
Serial PET-CT Scans Showing Lymphoma Involving the Myocardium in Patient 1 **(A)** Positron emission tomography–computed tomography (PET-CT) scan of Patient 1 at baseline shows nodular hypermetabolic activity in the myocardium, bones, and liver. **(B)** Interval PET-CT scan after bridging therapy shows improvement in fluorodeoxyglucose uptake in the myocardium. **(C)** At 1 month after chimeric antigen receptor T-cell therapy, PET-CT scan shows a near complete metabolic response systemically (except for physiological myocardial uptake).

**Figure 2:**
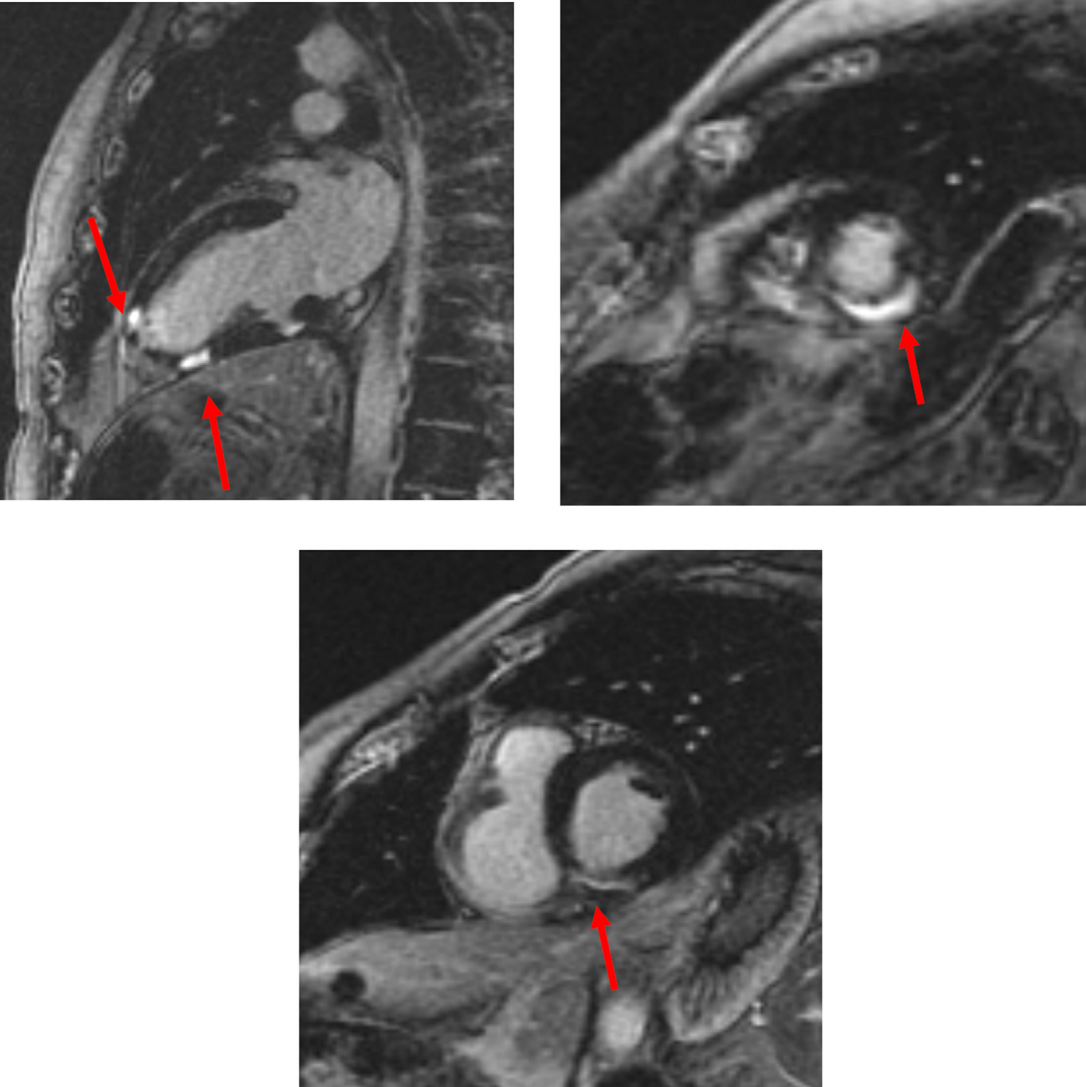
CMR Showing Lymphoma Involving the Myocardium in Patient 1 Cardiac magnetic resonance (CMR) shows patchy regions of mid myocardial/transmural foci of delayed enhancement involving the anterior, septal and inferior walls of the left ventricle **(red arrows)**, corresponding to site of lymphomatous involvement on positron emission tomography–computed tomography imaging.

**Figure 3:**
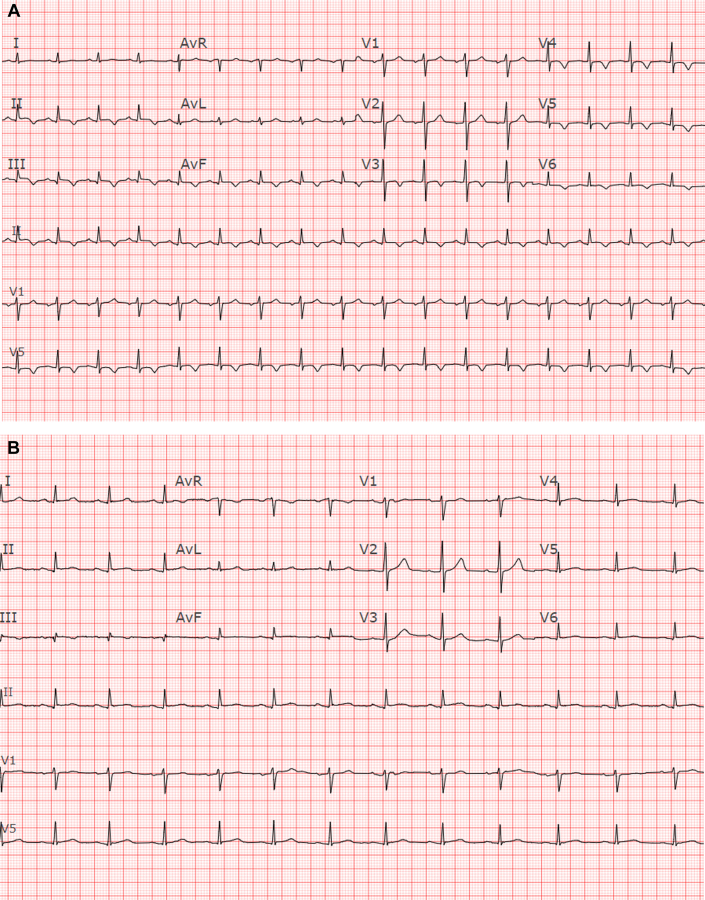
ECG of Patient 1 With Lymphoma **(A)** Resting electrocardiogram (ECG) of Patient 1 with lymphoma involving the myocardium before chimeric antigen receptor T-cell (CAR-T) therapy. ECG shows sinus tachycardia with a ventricular rate of 104 beats/min per minute and T-wave inversions in the inferior and inferolateral leads before CAR-T therapy. **(B)** Resting ECG post–CAR-T therapy in Patient 1. ECG shows resolution of tachycardia (sinus rhythm with a heart rate of 77 beats/min) and improvement in the inferior and inferolateral T-wave inversions post–CAR-T therapy.

A 37-year-old man with an initial diagnosis of follicular lymphoma that transformed into diffuse large B-cell lymphoma (DLBCL), despite multiple lines of chemotherapy and autologous stem cell transplantation (ASCT), presented for consideration of CAR-T therapy due to progressive disease. Fluorodeoxyglucose positron emission tomography–computed tomography (FDG PET-CT) and cardiac CT imaging revealed nodular hypermetabolic uptake and thickened myocardium (Figure 1A). This corresponded to areas of myocardial delayed enhancement (Figure 2) on cardiac magnetic resonance (CMR) (Figure 2, Videos 1A and 1B), consistent with lymphomatous myocardial involvement without suggestion of myocarditis. He had a preserved left ventricular ejection fraction (LVEF) of 59% on transthoracic echocardiogram (TTE). Resting electrocardiogram (ECG) showed sinus tachycardia and inferior and inferolateral T-wave inversions (Figure 3A). CT cardiac and angiogram imaging excluded both pulmonary embolism and obstructive coronary artery disease (Videos 2A and 2B). Troponin T levels were elevated with a peak level of 1,019 ng/L (normal, <15 ng/L), likely due to lymphomatous infiltration of the myocardium. The extent of lymphomatous cardiac involvement and overall disease burden decreased (Figure 1B) after 3 cycles of bridging therapy with rituximab, gemcitabine, dexamethasone, and cisplatin, and the patient made an informed decision to proceed with CAR-T therapy. After CAR-T infusion, the patient developed fever and hypotension consistent with grade 2 cytokine release syndrome (CRS) without cardiac decompensation. FDG PET-CT imaging 1 month post–CAR-T showed a near complete response systemically (Figure 1C). Sinus tachycardia and T-wave inversions also resolved on follow-up ECG (Figure 3B).

## Patient 2

**Figure 4:**
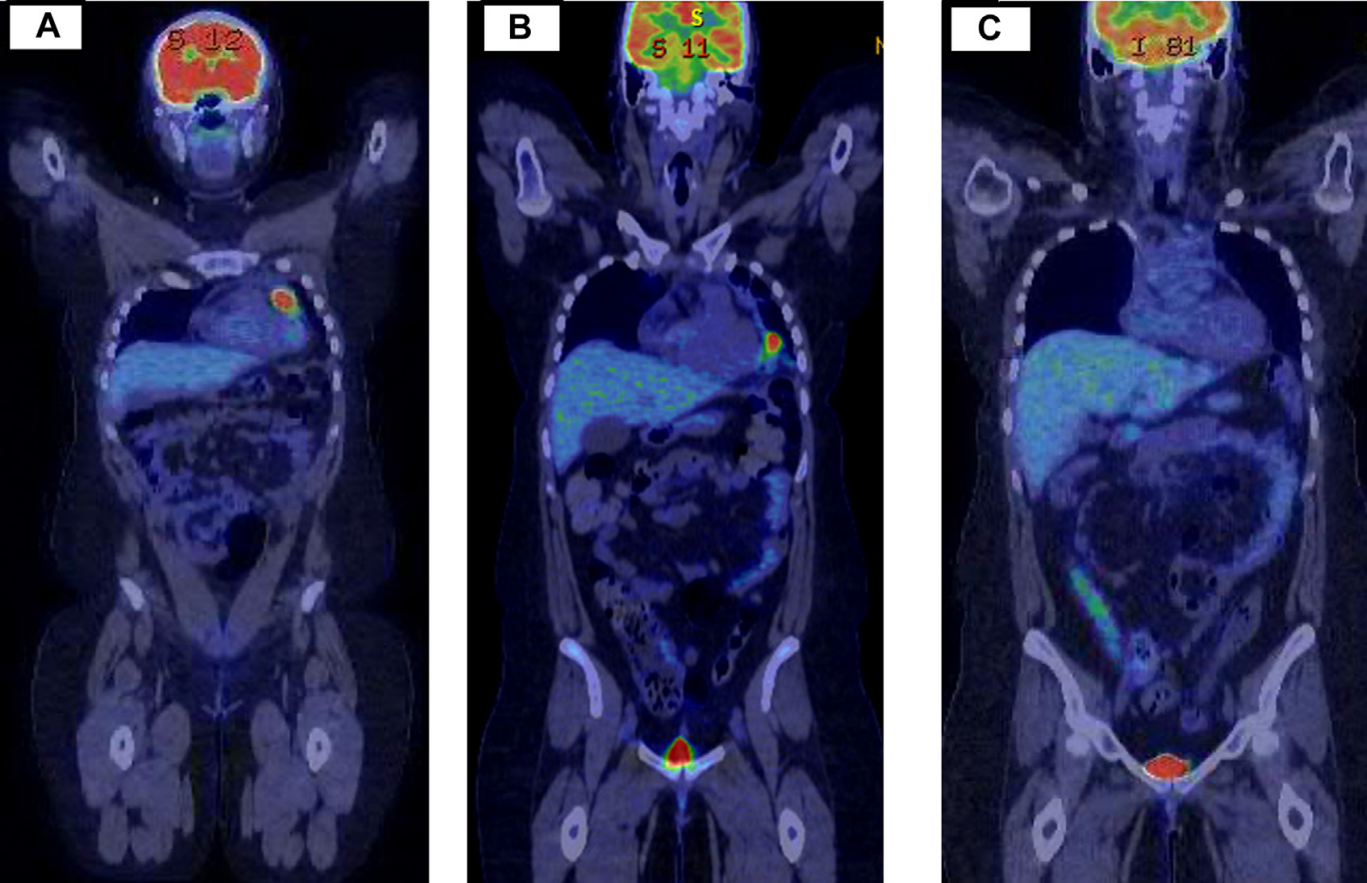
Serial PET-CT Scans Showing Lymphoma Involving the Pericardium in Patient 2 **(A)** PET-CT scan of Patient 2 at baseline shows a hypermetabolic mass involving the pericardium. **(B)** Interval PET-CT scan postsalvage therapy shows a response in the previously identified pericardial mass but with new areas of pericardial fluorodeoxyglucose activities. **(C)** At 1 month after CAR-T infusion, PET-CT scan shows improvement of fluorodeoxyglucose uptake in the pericardium. Abbreviations as in Figures 1 and 3.

**Figure 5:**
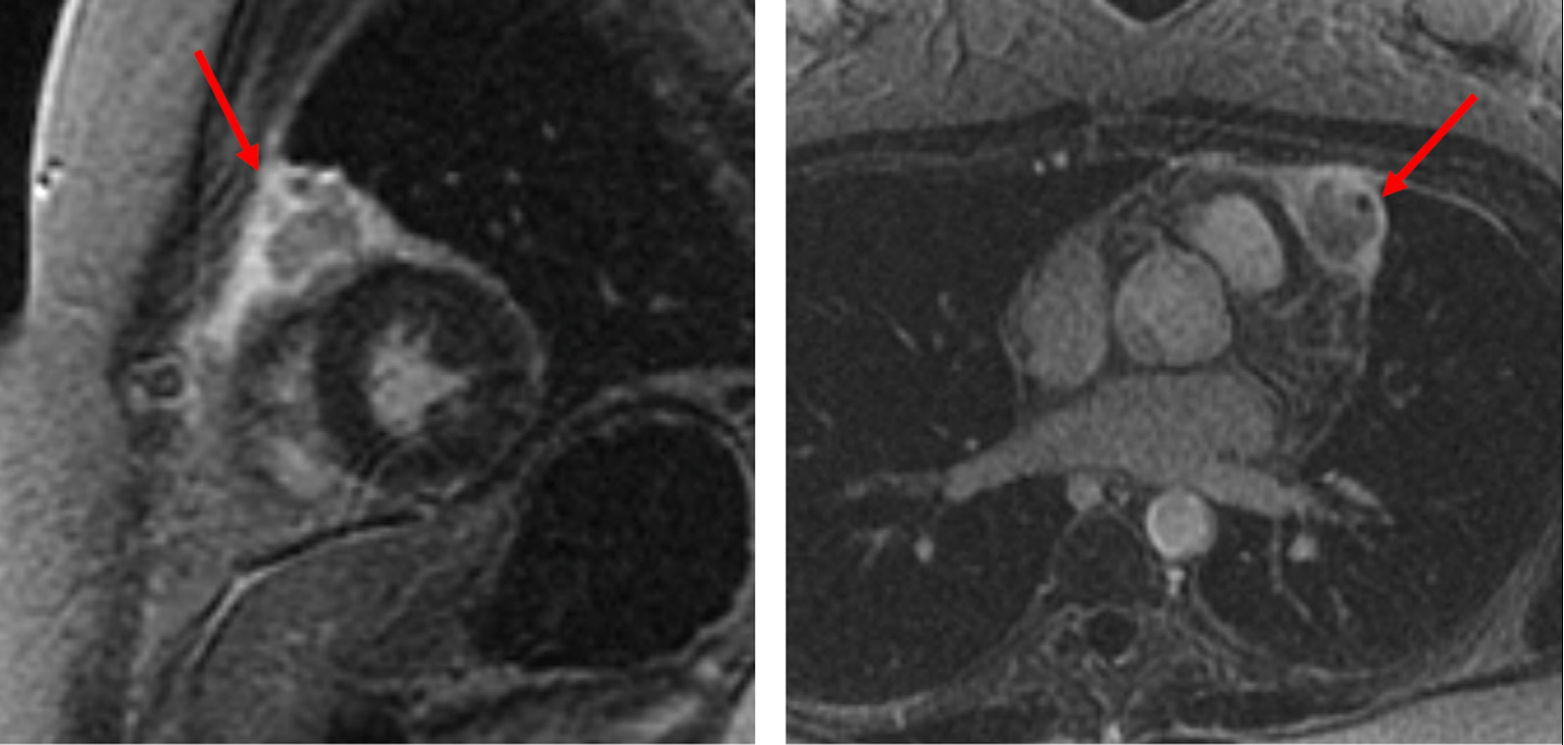
CMR Indicating Pericardial Involvement by Lymphoma in Patient 2 Cardiac magnetic resonance (CMR) indicates a pericardial/anterior mediastinal mass abutting the left ventricular apex with abnormal delayed enhancement, suggestive of lymphoma with pericardial involvement **(arrows in red)** in Patient 2.

A 30-year-old female patient with refractory primary mediastinal large B-cell lymphoma presented with chest pain, shortness of breath, and palpitations. FDG PET-CT imaging (Figure 4A) showed a hypermetabolic mediastinal mass involving the pericardium, and CMR (Figure 5, Video 3) revealed a soft tissue pericardial mass with abnormal delayed enhancement and heterogeneous perfusion consistent with lymphoma involving the pericardium. TTE revealed a normal LVEF of 65% without features of pericardial effusion or constrictive pericarditis. Interval PET-CT scan after salvage therapy (Figure 4B) showed less intensive FDG uptake in the pericardial mass. A multidisciplinary team including a cardio-oncologist and cardiothoracic surgeon discussed the risks of treatment such as constrictive pericarditis and pericardial perforation, and the patient proceeded with CAR-T therapy under telemetry monitoring. She developed hypotension, fever, and hypoxia consistent with grade 2 CRS requiring treatment with tocilizumab but did not have any clinical, electrocardiographic, or biochemical evidence of cardiac toxicity. Her 30-day follow-up FDG PET-CT scan showed improvement in FDG uptake involving the pericardium (Figure 4C).

## Patient 3

A 43-year-old male patient with DLBCL had disease progression despite multiple lines of chemotherapy and radiation therapy. The patient wished to proceed with CAR-T therapy even with a mildly reduced LVEF of 46% on TTE, likely related to his prior anthracycline exposure. Post–CAR-T therapy, the patient developed fever consistent with grade 1 CRS and did not have any cardiac decompensation. Cardiac biomarkers remained within normal limits. At 1-month follow up, FDG PET-CT imaging revealed slight progression of the disease. Although the 1-month post–CAR-T TTE showed a further decrease in LVEF to 41% (from 46%), the patient did not have any heart failure symptoms. He subsequently achieved complete remission of his lymphoma with immune checkpoint inhibition using pembrolizumab and did not experience any cardiac event.

## Patient 4

**Figure 6:**
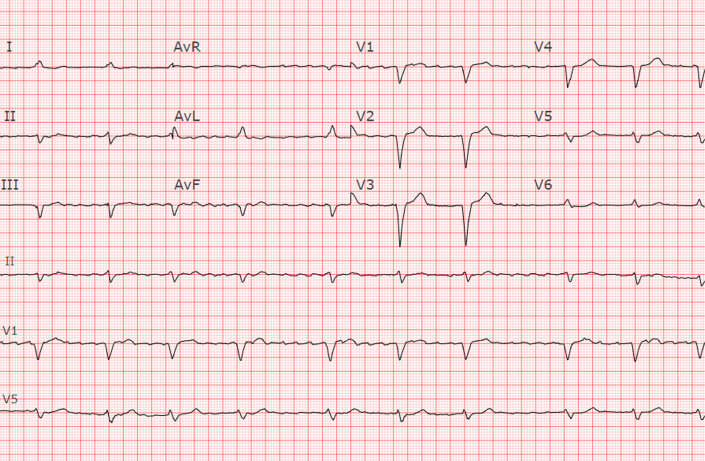
ECG Showing Atrial Flutter With Variable Block in Patient 4 Before CAR-T Therapy ECG showing atrial flutter with variable block with a ventricular rate of 58 beats/min, left-axis deviation, and left bundle branch block pattern. Abbreviations as in Figure 3.

A 66-year-old male patient developed nodal marginal zone lymphoma at the age of 32 years that was treated with chemotherapy and radiation therapy. More than 2 decades later, he developed DLBCL requiring further chemotherapy. Treatment was complicated by pulmonary embolism and congestive cardiac failure. He subsequently presented with bilateral pedal edema, and TTE confirmed a severely reduced LVEF of 20% to 25%. The cardiomyopathy was deemed secondary to the patient’s previous anthracycline exposure. After starting guideline-directed medical therapy with an angiotensin-converting enzyme inhibitor and beta-blocker, the patient’s LVEF improved to 35% to 40% on the repeat TTE 4 months later. He received additional non-anthracycline–based chemotherapy and underwent ASCT. A second relapse of DLBCL was noted 2 years later after a biopsy and histopathologic evaluation of a suspicious left lung lesion on surveillance FDG-PET CT imaging. Suitability of CAR-T therapy was discussed in a multidisciplinary forum. A dobutamine stress TTE pre–CAR-T therapy revealed a LVEF of 45% at rest and of 55% at peak stress without regional wall motion abnormalities, consistent with a normal contractile response and no evidence of ischemia. He also had new-onset atrial flutter (Figure 6), for which he received anticoagulant therapy and was started on amiodarone before CAR-T therapy. He proceeded with CAR-T therapy and developed grade 2 CRS (fever and hypotension), which was managed with tocilizumab and dexamethasone. The repeat TTE 30 days post–CAR-T indicated a drop in LVEF to 30% to 35% (from 45% before CAR-T therapy). Fortunately, he did not experience clinical heart failure or palpitations. Due to the decline in LVEF and recent-onset atrial flutter, cardioversion was attempted but remained unsuccessful. The patient declined catheter ablation and other measures. The repeat FDG PET-CT imaging on day 30 showed an interval metabolic response with marked reduction in the size of the lymphomatous mass in the left lower lung lobe.

## Patient 5

A 70-year-old female patient with IgA kappa multiple myeloma underwent chemotherapy, external beam radiation to both arms, and ASCT. She developed recurrent, progressive disease requiring a second ASCT and subsequent maintenance therapy with pomalidomide. TTE performed before her second ASCT showed a mildly reduced LVEF of 44%, which was attributed to a prior viral myocarditis. After starting guideline-directed medical therapy with a beta-blocker, the patient’s LVEF improved to 51%. Despite aggressive treatment, she experienced a myeloma relapse and was started on daratumumab and dexamethasone. She was also considered for CAR-T therapy. After a multidisciplinary discussion involving cardio-oncologists, the patient proceeded with CAR-T therapy. She developed fever and hypotension consistent with grade 2 CRS requiring tocilizumab and steroids, without any sign or symptom of heart failure. The repeat 1-month PET-CT scan showed a positive treatment response and marked improvement in FDG uptake.

## Discussion

CAR-T therapy has emerged as an effective treatment over recent years, especially for selected patients with relapsed or refractory B-cell lymphomas. Because seminal trials excluded patients with cardiac lymphoma or pre-existing cardiomyopathy, there is limited evidence for using CAR-T therapy in these patients.^1^, ^2^, ^3^, ^4^, ^5^, ^6^ In our case series, cases 1 and 2 illustrate patients with lymphoma and myocardial and pericardial involvement, respectively. Lymphomatous cardiac involvement has been reported in 8% to 20% of patients at autopsy.^7^ The patients described in cases 3 to 5 had coexisting cardiomyopathy. None of these patients experienced cardiovascular morbidity or mortality while undergoing CAR-T therapy.

Cardiotoxicity along with neurotoxicity are the most common complications seen with CAR-T therapy after CRS and can develop simultaneously in the same patient.^8^ Among patients receiving CAR-T therapy, approximately 57% to 93% of them will experience CRS, which typically presents as fever, hypotension, hypoxia, and/or multiorgan toxicity.^9^ Development of cardiotoxicity is likely caused by the proinflammatory milieu due to CAR-T therapy in the tumor microenvironment.^10^ Higher grades of CRS have been associated with cardiac complications, mainly arrhythmias, heart failure, and troponin elevation.^9^^,^^10^ Around 10% of patients undergoing CAR-T therapy may develop cardiomyopathy in the setting of high-grade CRS, and almost one-half of them have persistent cardiac dysfunction.^4^

As described in our case series, even patients with pre-existing cardiomyopathy, defined as an LVEF <50%, and those with cardiac lymphomatous involvement can be safely managed and undergo CAR-T therapy without significant cardiac adverse events. Dobutamine stress echocardiography could be used in pre–CAR-T therapy planning to assess contractile reserve, which is important under chronic stress conditions. Optimization of guideline-directed medical therapy before and continuing it during CAR-T therapy can be considered, while avoiding hypotension. In addition, cautious monitoring during CAR-T therapy and early treatment of CRS are pivotal.^10^

Likewise, patients with lymphomas affecting the myocardium or pericardium have historically been excluded from receiving CAR-T therapy.^2^, ^3^, ^4^^,^^6^ The concern has been that aggressive lysis of tumor cells in the myocardium could cause excessive inflammation and destabilization of anatomical structures, potentially leading to cardiac tamponade or fatal myocardial perforation. In the 2 at-risk cases outlined here, none of these complications occurred. It is important to emphasize that additional chemotherapy was given to reduce the burden of cardiac involvement before CAR-T therapy. All patients undergoing CAR-T therapies should have routine blood pressure monitoring; in patients with grade 2 or worse CRS or cardiac symptoms, obtaining TTE, ECG, and cardiac biomarkers could be useful to monitor for cardiotoxicity.^4^^,^^10^ In addition, a multidisciplinary approach with shared decision-making would be pivotal in managing such patients.

## Funding Support and Author Disclosures

Dr Y. Lin has received consultancy fees from Kite/Gilead, Celgene/BMS, Juno/BMS, bluebird bio, Janssen, Legend BioTech, Gamida Cells, Novartis, Iovance, Takeda, Fosun Kite, and Pfizer. Dr Herrmann has received consultancy fees from Pfizer and is supported by the National Cancer Institute (CA 233610). All other authors have reported that they have no relationships relevant to the contents of this paper to disclose.

## Appendix

Video 1A Short-axis cardiac magnetic resonance cine images covering the basal portion of the left and right ventricles demonstrating a preserved left ventricular ejection fraction of 59% with hypokinesis of the anterior, septal and inferior walls, suggestive of lymphomatous involvement of the myocardium in Patient 1. Short axis basal CMR cine images of patient 1 demonstrating hypokinesis of the anterior, septal and inferior walls, concerning for lymphomatous myocardial involvement.

Video 1B Short-axis cardiac magnetic resonance cine images covering the apical portion of the left and right ventricles demonstrating a preserved left ventricular ejection fraction of 59% with hypokinesis of the anterior, septal, and inferior walls, suggestive of lymphomatous involvement of the myocardium in Patient 1. Short axis apical CMR cine images of patient 1 demonstrating hypokinesis of the anterior, septal and inferior walls, concerning for lymphomatous myocardial involvement.

Video 2A Cardiac computed tomography short-axis views covering the basal portion of the left and right ventricles, showing focal areas of hypoenhancement, increased wall thickness, and hypokinesis in the inferior and inferoseptal walls at the base and mid ventricular level and involving the entire apex. This correlates with areas of increased fluorodeoxyglucose uptake in the heart on positron emission tomography–computed tomography imaging in Figure 1, consistent with lymphomatous involvement of the myocardium in Patient 1. Short axis basal cardiac CT views of patient 1 demonstrating focal areas of hypoenhancement, increased wall thickness, and hypokinesis in the inferior and inferoseptal walls correlating with areas of increased FGD uptake in the heart on PET-CT in figure 1, consistent with lymphomatous myocardial involvement.

Video 2B Cardiac computed tomography short-axis views covering the apical portion of the left and right ventricles, showing focal areas of hypoenhancement, increased wall thickness, and hypokinesis in the inferior and inferoseptal walls at the base and mid ventricular level and involving the entire apex, correlating with areas of increased fluorodeoxyglucose uptake in the heart on positron emission tomography–computed tomography imaging in Figure 1, consistent with lymphomatous involvement of the myocardium in Patient 1. Short axis apical cardiac CT views of patient 1 demonstrating focal areas of hypoenhancement, increased wall thickness, and hypokinesis, correlating with areas of increased FGD uptake in the heart on PET-CT in figure 1, consistent with lymphomatous myocardial involvement.

Video 3 Short-axis cardiac magnetic resonance showing a pericardial/anterior mediastinal mass abutting the left ventricular apex of the heart in Patient 2. The mediastinal mass exhibited heterogeneous perfusion. Short axis CMR views of patient 2 demonstrating a pericardial/anterior mediastinal mass with heterogenous perfusion, abutting the left ventricular apex of the heart.
