# Supplementary material for: CAR-T Therapy in Lymphoma Patients With Coexisting Cardiomyopathy or Cardiac Lymphomatous Involvement
Source: JACC Case Rep. 2023 Apr 21;15:101840. doi: 10.1016/j.jaccas.2023.101840 (PMC10240233; doi:10.1016/j.jaccas.2023.101840)
Supplement: Supplemental Table 1 [file mmc6.docx]

Supplemental Table 1: Summary of diagnosis, chemotherapeutic regimen, cardiac comorbidity, CRS and outcome of patients

| **Case** | **Demographics** | **Hematological Diagnosis** | **Treatment before CAR-T** | **Cardiac comorbidity** | **Type of CAR-T** | **Grading and treatment of peak Cytokine release syndrome (CRS) and Neurotoxicity** | **Cardiac outcome** |
| --- | --- | --- | --- | --- | --- | --- | --- |
| 1 | 37-year-old arabic male | Follicular lymphoma transformed into DLBCL with myocardial involvement | R-CHOP, R-ESHAP, R-ICE, ASCT | Myocardial involvement by lymphoma | Axicabtagene ciloleucel | Grade 2 CRS (Hypotension and fever).  Grade 1 neurotoxicity (Tremor, mentation and behavioral changes, and some difficulty with counting)  Did not require tocilizumab or steroids. | Improvement of FDG-PET uptake in the myocardium and stable mid myocardial foci of delayed enhancement on CMR which could represent treated lymphoma. |
| 2 | 30-year-old white female | DLBCL with pericardial involvement | EPOCH-R, R-ICE, R-CHOP, R-DHAP | Pericardial involvement by lymphoma | Axicabtagene ciloleucel | Grade 2 CRS (Fever, hypotension).  No neurotoxicity. Required tocilizumab. | Improvement in PET FDG uptake involving the pericardium. CMR showed stable size of anterior mediastinal and pericardial mass which extends from the right AV groove to the LV apex. |
| 3 | 43-year-old white male | DLBCL | R-CHOP, R-DHAP, R-ICE, radiation. | Pre-existing cardiomyopathy, likely related to previous anthracycline, LVEF of 46% on TTE. | Axicabtagene ciloleucel | Grade 1 CRS (Fever)  No neurotoxicity.  Did not require tocilizumab or steroids. | 1-month post CAR-T follow up FDG PET CT showed mixed response with overall disease progression of disease.  Repeat TTE 1-month after CAR-T showed a drop in LVEF from 46% to 41%. He did not have any clinical cardiac decompensation. |
| 4 | 66-year-old white male | Nodal marginal zone lymphoma/DLBCL | R-CHOP, R-ICE, ASCT | Anthracycline- related cardiomyopathy (LVEF of 20-25% with partial recovery to LVEF of 45% on goal-directed medical therapy), PE/DVT, new-onset atrial flutter, hyperlipidemia. | Lisocabtagene maraleucel | Grade 2 CRS (Fever and hypotension).  No neurotoxicity.  Required tocilizumab and steroids. | 1-month post CAR-T FDG PET showed marked partial response. Repeat TTE 1-month post CAR-T demonstrated drop in LVEF to 35-40% (from 45% pre CAR-T). |
| 5 | 70-year-old white female | Multiple myeloma | VRd, KPd, ASCT | Tobacco. Cardiomyopathy likely related to previous viral myocarditis with partial recovery of LVEF from 44% to 51%. | Ciltacabatagene Autoleucel | Grade 2 CRS (Fever and hypotension)  No neurotoxicity.  Required tocilizumab and steroids. | 1-month post CAR-T FDG PET showed positive interval treatment response with marked improvement in FDG avid lesions. No post CAR-T TTE |

ASCT: Autologous stem-cell transplantation; CAR-T: Chimeric antigen receptor T-cell; CMR: Cardiac magnetic resonance; CRS: Cytokine release syndrome; DLBCL: Diffuse large B-cell lymphoma; EPOCH-R: Etoposide, prednisone, vincristine, cyclophosphamide, doxorubicin, and rituximab; GDP: Gemcitabine, dexamethasone, and cisplatin; KPd: Carfilzomib-Pomalidomide-Dexamethasone; R-CHOP: Rituximab, cyclophosphamide, doxorubicin, vincristine, and prednisone; R-DHAP: Rituximab. dexamethasone, cytarabine, cisplatin; R-ESHAP: Rituximab, etoposide, methylprednisone, cytarabine, and cisplatin; R-ICE: Rituximab, ifosfamide, carboplatin, and etoposide.
